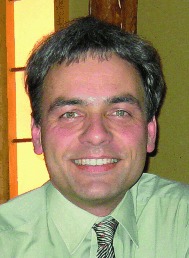# Opening Up the World of Chemistry

**DOI:** 10.1002/open.201100009

**Published:** 2012-01-02

**Authors:** Ramón Martínez-Máñez, Thomas Wirth

**Affiliations:** Polytechnic University of ValenciaSpain; Cardiff UniversityUnited Kingdom

…knowing that numerous European Chemical Societies support the development of *ChemistryOpen*, it became apparent that this would be a distinctive project and an exciting opportunity.

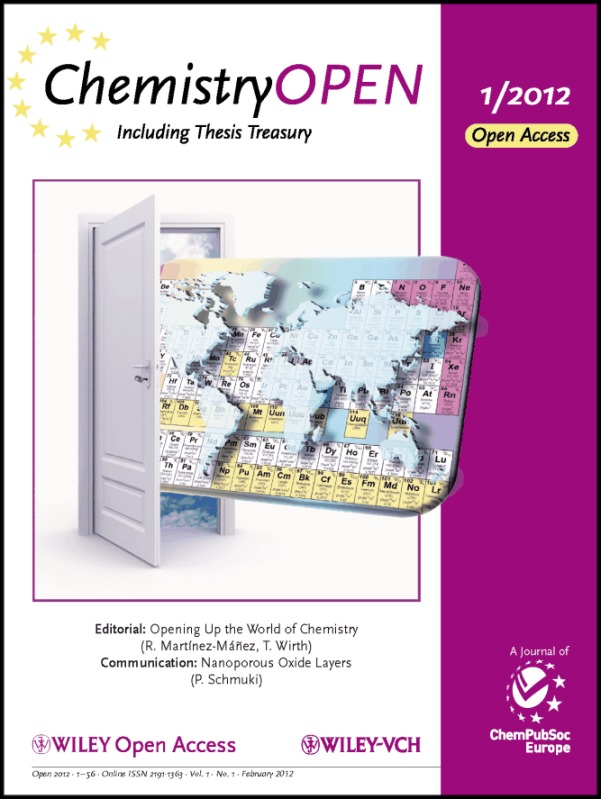
 Not too long ago, we received an invitation from Wiley-VCH to be co-chairmen of a new open-access journal: *ChemistryOpen*. We had both previously received similar invitations to join the boards of other open-access journals but declined these offers; however, after reading the letter from Wiley-VCH and knowing that numerous European Chemical Societies support the development of *ChemistryOpen*, it became apparent that this would be a distinctive project and an exciting opportunity.

Discussions concerning open-access publishing are ongoing. However, there are many good reasons why gold-road, open-access journals such as *ChemistryOpen* can fill a gap and co-exist alongside traditional scientific journals. Greater access to research outputs will allow academics and commercial companies to benefit directly through increased innovation and shorter development cycles. In addition, several government funding agencies nowadays require open-access publications of the research they have sponsored. Indeed, Wiley–Blackwell recently announced open-access agreements with and the Max Planck Society in Germany, the FWF Austrian Science Fund, and Telethon, one of the largest biomedical non-profit organizations in Italy. Research results paid by public funds should not be locked away in journals only available to subscribers. ChemPubSoc Europe, a group of 16 continental European chemical societies, was concerned with these issues and aware of the fact that, at that time, no chemical society published an open-access journal. With this background, *ChemistryOpen*—an open-access, international, multidisciplinary chemistry journal—was born, targeted to a broad and general readership, and aiming for the publication of high-level Full Papers and Communications from all areas of chemistry.


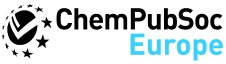
 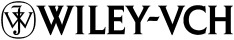 
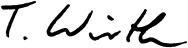
 
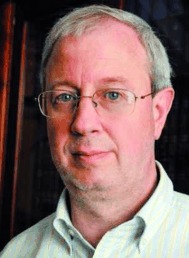


In addition, *ChemistryOpen* contains a Thesis Treasury, which offers the unique possibility to publish PhD thesis summaries, with the full version archived as Supporting Information and available via Wiley Online Library. These summaries are given digital object identifiers (DOIs) to facilitate citation and make the material “discoverable”. The fact is that not all scientific research is published, searchable or available electronically, and the Thesis Treasury aims to address this disparity. Of course, finding the information is now easier, but reading is still required!

We expect *ChemistryOpen* will soon reach the very high-standards of other general chemistry journals published by Wiley-VCH…

We expect *ChemistryOpen* will soon reach the very high-standards of other general chemistry journals published by Wiley-VCH, such as *Angew. Chem. Int. Ed*., *Chem. Eur. J*. and *Chem. Asian J*. With this aim, we have joined *ChemistryOpen* as Co-chairmen of the Editorial Advisory Board, and we are excited about the possibility to contribute to this remarkable and unique project.

We hope that the accessing of interesting new scientific results is now made easier for researchers with the launch of *ChemistryOpen.* We also hope you enjoy this inaugural issue and that you will continue to consult *ChemistryOpen* as an essential source of current and advanced developments in chemistry. Finally, we thank all of the authors that have contributed to this first issue. We look forward to receiving your manuscript at *ChemistryOpen*, and assure you that it will benefit from the open access and easy sharing with the whole scientific community.